# Ultrasonic-Assisted Enzymolysis Extraction and Protective Effect on Injured Cardiomyocytes in Mice of Flavonoids from *Prunus mume* Blossom

**DOI:** 10.3390/molecules26195818

**Published:** 2021-09-25

**Authors:** Shengnan Zhu, Jicheng Xu, Huizhi Chen, Weiqiao Lv

**Affiliations:** 1 College of Biological and Food Engineering, Anhui Polytechnic University, Wuhu 241000, China; shengnanzhu1@163.com; 2 State Key Laboratory of Food Science and Technology, Jiangnan University, Wuxi 214122, China; chenhuizhiht@163.com; 3 College of Engineering, China Agricultural University, Beijing 100083, China; lvweiqiao@cau.edu.cn

**Keywords:** ultrasonic-assisted, enzymolysis extraction, *Prunus mume* blossom, immune regulation

## Abstract

*Prunus mume* blossom is an edible flower that has been used in traditional Chinese medicine for thousands of years. Flavonoids are one of the most active substances in *Prunus mume* blossoms. The optimal ultrasonic-assisted enzymatic extraction of flavonoids from *Prunus mume* blossom (FPMB), the components of FPMB, and its protective effect on injured cardiomyocytes were investigated in this study. According to our results, the optimal extraction process for FPMB is as follows: cellulase at 2.0%, ultrasonic power at 300 W, ultrasonic enzymolysis for 30 min, and an enzymolysis temperature of 40 °C. FPMB significantly promoted the survival rate of cardiomyocytes and reduced the concentration of reactive oxygen species (ROS). FPMB also improved the activities of proteases caspase-3, caspase-8, and caspase-9 in cardiomyocytes. The cardiomyocyte apoptosis rate in mice was significantly reduced by exposure to FPMB. These results suggest that the extraction rate of FPMB may be improved by an ultrasonic-assisted enzymatic method. FPMB has a protective effect on the injured cardiomyocytes.

## 1. Introduction

*Prunus mume* is an edible flower used in traditional Chinese medicine. It is commonly used to prevent and treat various infections and inflammation, having antibacterial and anti-inflammatory effects [1]. *Prunus mume* blossoms have high medicinal value and health care functions. Flavonoids are some of the most active substances in *Prunus mume* blossoms. As a natural product mainly found in plants, flavonoids have many important physiological and biochemical effects due to their unique chemical structure [2]. The active ingredients of many traditional Chinese medicinal materials are flavonoids [3,4]. Flavonoids improve the blood circulation of the human body and are a strong antioxidant, effectively eliminating free radicals in the body with a protective effect on cell aging and degradation [5,6,7].

China is one of the major countries in the production and utilization of *Prunus mume* blossom [8]. The extraction, identification, and use of flavonoids from the *Prunus mume* blossom are of great significance. An enzyme solution was used to damage the cell walls of the plant tissue when the flavonoids were extracted, thereby improving the effectiveness of the extraction [9,10,11]. The extraction rate of flavonoids improved after adding enzymes into the ground *Prunus mume* blossom powder. Enzymic extraction provides the advantages of simple operation and mild reaction conditions. However, the sheer force extracted by the enzyme method is insufficient to destroy the plant cell wall, causing a low extraction rate [12,13]. An ultrasonic wave is a type of mechanical wave with a short wavelength. Ultrasound can break or dissolve cell walls and membranes, releasing flavonoids from the plants [14,15,16].

Therefore, the purpose of this study was to use ultrasound to enhance the enzymatic extraction effect of flavonoids from *Prunus mume* blossom (FPMB). The components of FPMB were identified by high-performance liquid chromatography (HPLC). Furthermore, we studied the effects of FPMB on cardiomyocyte activity; the concentrations of reactive oxygen species (ROS); the concentrations of proteases caspase-3, caspase-8, and caspase-9; and the cardiomyocyte apoptosis of mice. These results provide a reference for the extraction and biological activity of FPMB.

## 2. Results and Discussion

### 2.1. Single-Factor Test Results

At a temperature of 40 °C, ultrasonic power of 300 W, and ultrasonic time of 40 min, cellulase with the mass percentage of 0.5%, 1.0%, 1.5%, 2.0%, 2.5%, and 3.0% was added to conduct the extraction experiment of FPMB. The results are shown in Table 1. Under the conditions of 2.0% cellulase, 300 W ultrasonic power, and 40 min ultrasonic time, the extraction experiments of FPMB were conducted at the hydrolysis temperatures of 30, 35, 40, 45, 50, and 55 °C; the results are shown in Table 1. The increase in cellulase mass percentage and the extraction rate of FPMB gradually increased (Table S1). The reason for this increase may be that cellulase destroys cell walls, leading to a significant increase in the extraction rate of flavonoids. However, when the cellulase mass percentage were 2.0%, 2.5%, and 3.0%, the extraction rate of FPMB showed no significant difference. This insignificant difference may be because the cell wall in the *Prunus mume* blossom fully dissolves when the cellulase mass percentage reaches 2.0%. The extraction rate of FPMB was the highest at the temperature of 40 °C. The extraction rate of flavonoids did not significantly improve when the temperature continued to increase. The reason for this phenomenon may be that the effect of 40 °C on the extraction rate of flavonoids reached the maximum.

Under the conditions of 2.0% cellulase, 40 °C hydrolysis temperature, and 40 min of ultrasonic time, the FPMB extraction experiments were conducted at the ultrasonic power of 200, 250, 300, 350, 400, and 450 W. The results are shown in Table 2. Under the conditions of 2.0% cellulase, 40 °C hydrolysis temperature, and 300 W ultrasonic power, the FPMB extraction experiments were conducted at the ultrasonic times of 20, 30, 40, 50, 60, and 70 min. The results are shown in Table 2. As can be seen from the table, with the increase in ultrasonic power, the extraction rate of FPMB gradually increased (Table S1). However, when the ultrasonic power reached 300 W, the extraction rate of FPMB no longer increased and trended downward. This may be because the ultrasonic power was too high, destroying some of the flavonoids. The extraction rate for FPMB gradually increased with the increase in ultrasonic enzymolysis time. However, when the ultrasonic enzymolysis time surpassed 40 min, the extraction rate of FPMB did not change; this may be because the effect of ultrasonic enzymolysis time on the extraction rate of FPMB reached the maximum.

### 2.2. Orthogonal Test Results

According to the single-factor test results, cellulase concentration, enzymolysis temperature, ultrasonic power, and ultrasonic-assisted enzymolysis time were considered as factors. Each factor was designed with three levels. The concentration of the cellulase was 1.5%, 2.0%, or 2.5%. The enzymolysis temperature was 35, 40, or 45 °C. The ultrasonic power was 250, 300, or 350 W. The ultrasonic-assisted enzymolysis time was 30, 40, or 50 min, respectively. The test results are shown in Table 3, and the variance analysis results are shown in Table 4.

As seen in Table 3, among the different single factors, the degree of influence on the extraction rate of FPMB was: cellulase concentration > ultrasonic power > enzymolysis temperature = ultrasonic-assisted enzymolysis time. Therefore, the optimal technological conditions for the ultrasonic-assisted enzymolysis extraction of FPMB were A_3_B_3_C_3_D_2_. Further analysis is needed to determine whether single factors have a significant influence on the extraction rate of FPMB.

Table 4 shows the results of the orthogonal test variance analysis. The concentration of cellulase and ultrasonic power had a significant impact on the extraction rate of FPMB. The effects of enzymolysis temperature and ultrasonic-assisted enzymolysis time on the extraction rate of FPMB were not significant. These results indicated that cellulase concentration and ultrasonic power were important factors in the range of every single factor. Combined with the previous range analysis results, from the energy-saving and time-saving perspectives, B_1_ was selected as the enzymolysis temperature and D_1_ as the ultrasonic-assisted enzymolysis time. Therefore, the optimal process for the ultrasonic-assisted enzymolysis extraction of FPMB determined by orthogonal experiments was A_3_B_1_C_3_D_1_; namely, the cellulase concentration was 2.0%, the temperature of enzymolysis was 40 °C, the ultrasonic power was 300 W, and the time of ultrasonic-assisted enzymolysis hydrolysis was 30 min.

### 2.3. Identification of Main Components in FPMB

**Figure 1:**
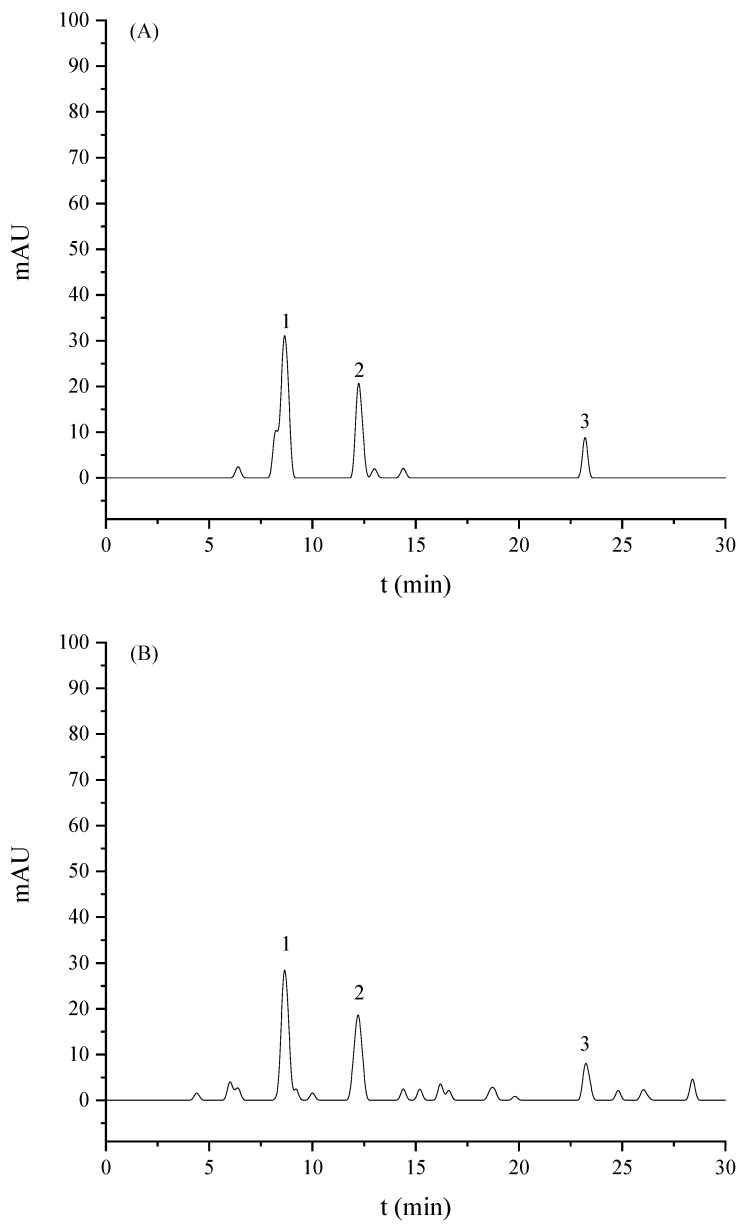
HPLC chromatograms of mixed standards (**A**) and FPMB (**B**).

As shown in Figure 1, FPMB is mainly composed of three compounds. The retention time of the three compounds was 8.60, 12.20, and 23.20 min, respectively. The peaks of these three compounds were consistent with those of rutin, cynarin, and luteolin by HPLC compared with mixed standard substances. The results showed that the main flavonoids in FPMB were rutin, cynarin, and luteolin.

FPMB is a kind of new flavonoid. There were several peaks in the HPLC chromatogram of the FPMB samples. The identification of flavonoid types in FPMB should be made using more specific techniques. In addition, the different habitats of *Prunus Mume* blossoms may lead to different types and mass percentage of FPMB. The separation and identification of these flavonoids are difficult and must be improved in the future.

### 2.4. Effect of FPMB on the Cardiomyocytes’ Survival Rate

**Figure 2:**
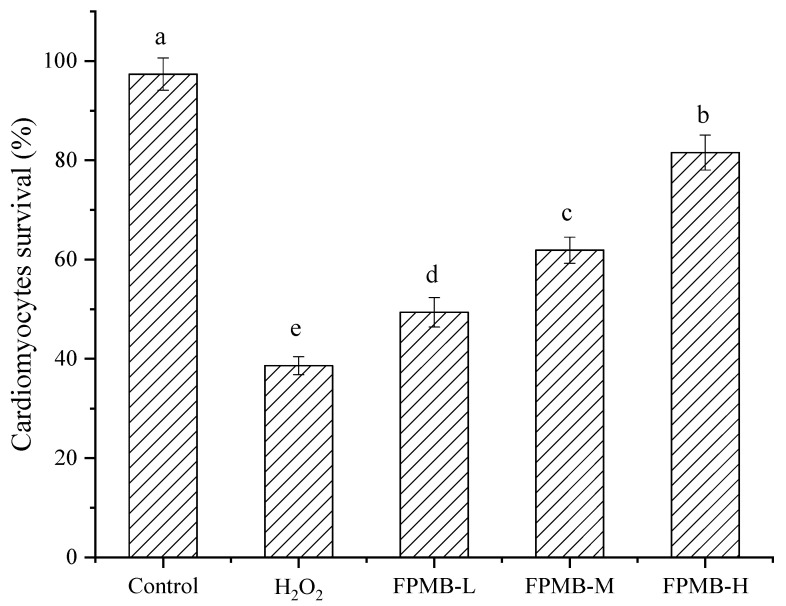
Effect of FPMB on the cardiomyocyte survival for mice injured by H_2_O_2_. Different lowercase letters (a, b, c, d, e) express significant differences (*p* < 0.05).

Figure 2 shows the effect of FPMB on the cardiomyocytes’ survival rate in mice injured by H_2_O_2_. The survival rate of cardiomyocytes in the model group was significantly lower than that in the control group (*p* < 0.05). However, the survival rate of cardiomyocytes in the three groups with FPMB was significantly higher than in the model group (*p* < 0.05). The survival rate of cardiomyocytes also increased significantly (*p* < 0.05) with the increase in FPMB dose. However, the survival rate of cardiomyocytes in the FPMB-H group did not return to the control group.

Cardiomyocytes are also known as cardiac fibers. All types of cardiomyocytes work together to maintain the complete function of the heart [17]. The measurement of cardiomyocytes activity is of great value for identifying the pathological process of heart disease [18]. In this study, H_2_O_2_ significantly reduced the viability of cardiomyocytes (*p* < 0.05). After the exposure of FPMB, the cardiomyocyte activity increased significantly, trending with the increase in FPMB dose. The results mentioned above indicated that FPMB has a protective effect on the cardiomyocytes injured by H_2_O_2_.

### 2.5. Effect of FPMB on the ROS Contents

**Figure 3:**
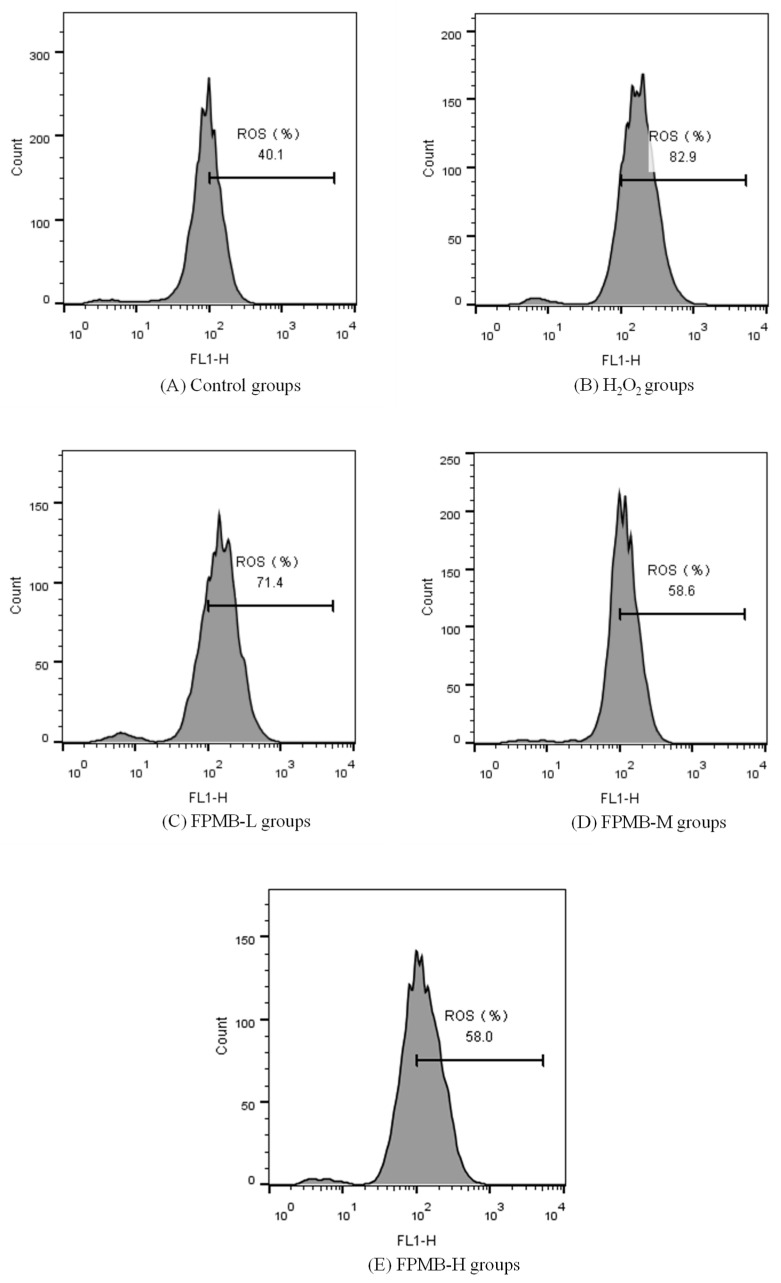
Effect of FPMB on the ROS contents of mouse cardiomyocytes injured by H_2_O_2_. (**A**) Control groups; (**B**) H_2_O_2_ groups; (**C**) FPMB-L groups; (**D**) FPMB-M groups; (**E**) FPMB-H groups.

Figure 3 shows the effect of FPMB on the ROS content of cardiomyocytes. The ROS content in the model group increased by 107% compared with the control group. This result indicated that under H_2_O_2_ induction, the balance of ROS production and clearance in cardiomyocytes was disturbed. ROS accumulated in large quantities in the cardiomyocytes. The ROS content of the cardiomyocytes gradually recovered after exposure FPMB. In the FPMB-H group, ROS contents of cardiomyocytes were reduced to 30% compared with the H_2_O_2_ group. This result indicated that FPMB effectively reduces the ROS content of cardiomyocytes when consumed.

ROS refers to a class of chemically active compounds containing oxygen groups with a strong oxidation ability that play an important role in cell signaling and homeostasis [19]. Under normal conditions, ROS production and clarity are balanced. However, during an emergency, ROS are produced in large quantities in cardiomyocytes. ROS damage the integrity of mitochondria and the cell membrane of cardiomyocytes, leading to oxidative stress damage of cell membranes and inducing apoptosis [20]. In this study, the use of hydrogen peroxide led to a significant increase in the ROS content of cardiomyocytes. When model mice were fed FPMB, the ROS content of myocardial cells decreased significantly. This study showed that FPMB has a protective effect on the H_2_O_2_-induced oxidative stress injury of cardiomyocytes.

### 2.6. Effect of FPMB on the Activities of Caspase-3, Caspase-8, and Caspase-9

**Figure 4:**
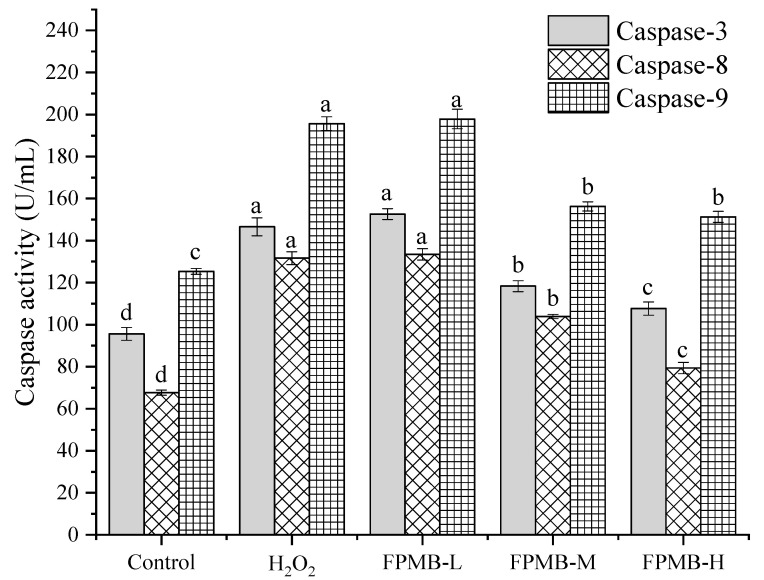
Effect of FPMB on the activity of caspase-3, caspase-8, and caspase-9 in mice cardiomyocytes injured by H_2_O_2_. Different lowercase letters (a, b, c, d) in the same caspase express significant differences (*p* < 0.05).

Figure 4 shows the effects of FPMB on the activities of caspase-3, caspase-8, and caspase-9 in the injured cardiomyocytes. The activities of cardiomyocytes caspase-3, -8, and -9 in the model group were significantly higher than those in the control group (*p* < 0.05). The activities of caspase-3, caspase-8, and caspase-9 in the FPMB-L group were not significantly different from those in the model group. This result showed that FPMB had no significant effect on the activities of cardiomyocytes caspase-3, caspase-8, and caspase-9 at this concentration. The activity of cardiomyocytes caspase-3, caspase-8, and caspase-9 was significantly lower than the model group (*p* < 0.05) when the concentration of FPMB increased; thus, a higher concentration of FPMB has a protective effect on injured cardiomyocytes induced by H_2_O_2_.

In 1994, Prins et al. [21] found that apoptosis occurs in normal adult adipocytes, revealing the existence of apoptosis in mature adipocytes for the first time. Numerous studies have since verified that the caspase family was essential in mediating apoptosis [22,23]. In the caspase family known currently, caspase-3, caspase-8, and caspase-9 are closely related to apoptosis. Caspase-8 and caspase-9 are important promoters of apoptosis, and caspase-3 is an important executor of apoptosis. Caspase-3, caspase-8, and caspase-9 all play key roles in the process of apoptosis [24]. Therefore, caspase-3, caspase-8, and caspase-9 were selected as research objects for this study. According to the results of our experiment, the activities of caspase-3, caspase-8, and caspase-9 in the FPMB groups were significantly decreased compared with those of the model group. The reason for this decrease may be due to FPMB reducing apoptosis of cardiomyocytes by decreasing caspase-3, caspase-8, and caspase-9 activities.

### 2.7. Effect of on Cardiomyocytes’ Apoptosis

**Figure 5:**
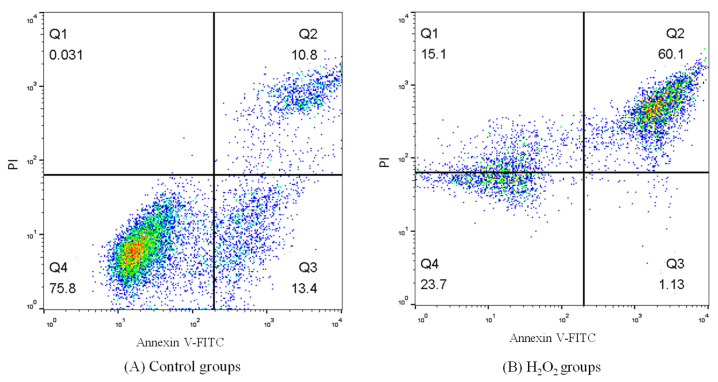

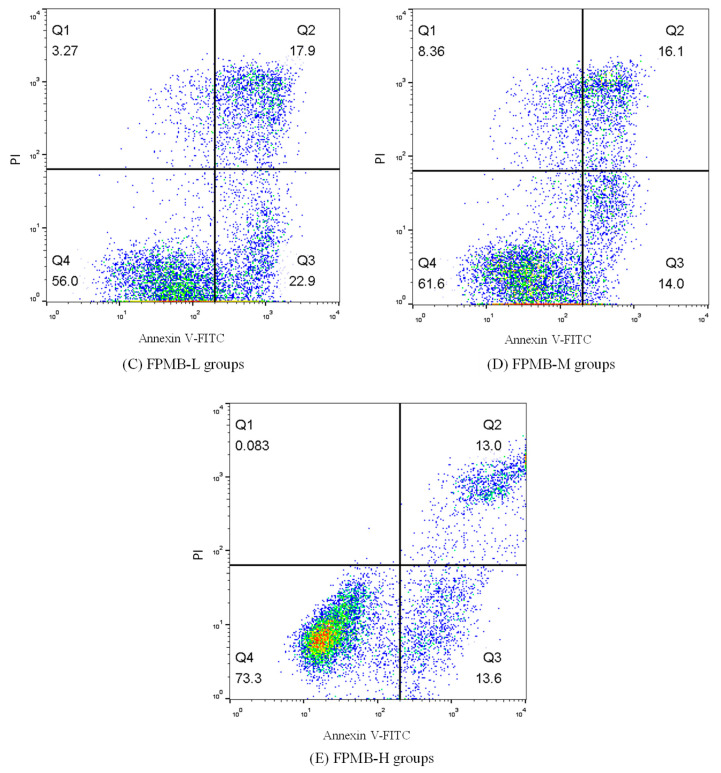
Effect of FPMB on the apoptosis of mice cardiomyocytes injured by H_2_O_2_. (**A**) Control groups; (**B**) H_2_O_2_ groups; (**C**) FPMB-L groups; (**D**) FPMB-M groups; (**E**) FPMB-H groups.

Table 5 and Figure 5 show the effects of FPMB on the apoptosis of injured cardiomyocytes. These results showed that the apoptosis rate of cardiomyocytes in the control group was only 24.20%. However, the apoptosis rate of the H_2_O_2_ model group reached 61.23%. Therefore, the oxidative damage model induced by H_2_O_2_ was successful. The apoptosis rate of cardiomyocytes decreased significantly after being treated with different concentrations of FPMB. The cardiomyocytes apoptosis rate decreased by 57% in the FPMB-H group compared with the H_2_O_2_ model group.

Apoptosis is a programmed and active mode of death regulated by genes under a normal physiological or pathological environment [25]. When external physical and chemical factors stimulate cells, apoptosis accelerates [26]. However, flavonoids alleviate the occurrence of apoptosis. In this study, the apoptosis rates of mouse cardiomyocytes in the FPMB group significantly reduced (*p* < 0.05) compared with the H_2_O_2_ model group. The apoptosis rate of cardiomyocytes was close to that of the control group with the increase in FPMB dose. These results showed that FPMB has a restorative effect on the apoptosis of cardiomyocytes.

## 3. Materials and Methods

### 3.1. Raw Materials

*Prunus mume* blossoms (1 year old, 99% purity) were purchased from Zhongshan Pharmacy in Wuhu, Anhui Province, China. The clean grade male mice (12 weeks old) were purchased from the Changzhou Cavins Laboratory Animal Co., Ltd. (Changzhou, China). Acidic cellulase (200,000 U/g) was purchased from Anhui Yinqiao Biotechnology Co., Ltd. (Shanghai, China). Caspase-3, caspase-8, and caspase-9 kits were provided by Seymour Fly Biochemistry Products Co., Ltd. (Shanghai, China). Hydrogen peroxide (H_2_O_2_), dimethyl sulfoxide (DMSO), Dulbecco′s modified eagle medium (DMEM), 3-(4,5-dimethylthiazole-2)-2,5-diphenyltetrazolium bromide (MTT), and fluorescent dye 2′,7′-dichlorodihydrofluorescein diacetate (DCFH-DA) were purchased from Bomer Biotechnology Co., Ltd. (Shanghai, China). All other reagents were analytically pure.

### 3.2. Extraction of FPMB

The *Prunus mume* blossom was dried using a microwave (600 W, 3–4 min) and ground to powder. Then, 10.0 g of *Prunus mume* blossom powder was accurately weighed for each group. Distilled water (200 mL) was added to each group. The FPMB was extracted with different cellulase mass percentages, enzymolysis temperatures, ultrasonic power, and ultrasonic enzymolysis time. The extract was vacuum lyophilized into powder. The freeze-dried powder was then configured into solutions of 10, 20, and 40 µg/mL for the cardiomyocytes protective effect test.

### 3.3. Measurements and Analyses

#### 3.3.1. Determination of Flavonoids Content

##### Drawing of a Standard Curve

The standard curve was drawn according to the method of Chen et al. [27]. When the mass concentration of rutin was in the range of 0.00–0.20 mg/mL, the absorbance showed a strong linear relationship with the mass concentration (Figure S1). The regression equation is: y = 8.7214x + 0.0629 (R^2^ = 0.9928), where y represents the OD value and X represents the concentration of rutin standard solution (mg/mL).

##### Flavonoids Content Measurement

In this test, sodium nitrite-aluminum nitrate colorimetry measured the flavonoids content. The sample liquid (5 mL) was absorbed by a pipette in each group, added into a 50 mL volumetric flask treated according to the above method, and stabilized at 50 mL with 75% ethanol. The absorbance of each test tube was measured at a wavelength of 510 nm. The content of flavonoids can be obtained from the regression equation [27].

(1) $$\mathrm{Extraction}\;\mathrm{rate}\;\mathrm{of}\;\mathrm{flavonoids}\;(100\%)=\frac{C\;\times \;N\;\times \;V\;}{M}\times 100\%\;$$

where C is the flavonoids content (mg/mL), N represents the dilution factor, V is the total volume (mL) of the extract solution, and M represents the quality of the *Prunus mume* blossom.

#### 3.3.2. Single-Factor Test Design

Each group of single-factor experiments used 10 g of *Prunus mume* blossom powder and 200 mL of deionized water. The single-factor test was conducted using cellulase mass percentage (1.5%, 2.0%, and 2.5%), enzymolysis temperatures (35, 40, and 45 °C), ultrasonic power (250, 300, and 350 W), and ultrasonic enzymolysis time (30, 40, and 50 min).

#### 3.3.3. Orthogonal Experimental Design

According to the results of the single-factor experiment, three levels were designed for each factor, including cellulase concentration, enzymolysis temperature, ultrasonic power, and ultrasonic enzymolysis time. An L_9_(3^4^) orthogonal experiment was designed with the extraction rate of FPMB powder as the index to optimize the technological conditions of ultrasonic-assisted enzymatic extraction.

#### 3.3.4. Identification of FPMB by HPLC

The FPMB lyophilized powder (5 mg) was accurately weighed. A sample solution of 1.0 mg/mL was prepared from the lyophilized powder of FPMB in a 10 mL volumetric flask. The sample solution and standard solution were diluted with 30% methanol 5 times. The diluted solution was filtered with an organic membrane of 0.5 µm. The filtered solution was then analyzed by HPLC (H-CLASS, Waters Technologies, Inc., Shanghai, China). The type of chromatographic column was ALPHA1-2 LD plus (Bio-labs Instruments Co., Ltd., Guangzhou, China). The chromatographic conditions included the mobile phase: methanol (A), ultra-pure water (B), and 1% acetic acid solution (C): flow rate: 1.0 mL/min; injection volume: 10 μL; elution conditions: 0–30 min, 30–80% A, 1% acetic acid solution (C) always maintained at 10%; detection wavelength: 350 nm; and column temperature: 30 °C.

#### 3.3.5. Isolation of Cardiomyocytes

Under aseptic conditions, mice were euthanized, soaked in 75% ethanol for 30 s, their chest opened with a scalpel, and their ventricles removed and rinsed with phosphate-buffered saline (PBS) 2–3 times [28]. The ventricles were cut into small pieces of about 1–1.5 mm^3^ in a petri dish. Afterward, 1 mL of trypsin (0.1%) was poured into the petri dish and digested for 10 min in a 37 °C water bath. After filtration with 200 mesh sieves, the mixture was centrifuged at 424× *g* for 8 min. The cardiomyocytes precipitation was added to DMEM (containing 15% fetal bovine serum) and cultured in a CO_2_ incubator for 2 h. The supernatant was obtained for counting. The cell density was diluted to 2 × 10^5^ mL^−1^. The cardiomyocyte suspension was inoculated on 96-well plates (100 µL per well) in a 37 °C CO_2_ incubator, and the culture medium was changed every 24 h. The cardiomyocytes in suitable growth conditions were selected for tests after 72 h.

#### 3.3.6. Design of Protective Effect Test on Cardiomyocytes

The well-grown cardiomyocytes were randomly divided into the control group, H_2_O_2_ model group, low-dose group (FPMB-L), medium-dose group (FPMB-M), and high-dose group (FPMB-H), with 10 replicates for each group. The control group was supplemented with 100 µL DMEM. The H_2_O_2_ model group was supplemented with 100 µL H_2_O_2_ (200 µmol/L). The FPMB-L, FPMB-M, and FPMB-H groups were supplemented with 100 µL DMEM medium (containing FPMB at concentrations of 10 µg/mL, 20 µg/mL, and 40 µg/mL, respectively) and H_2_O_2_ (200 µmol/L). After incubation for 12 h, cardiomyocytes’ activity, ROS content, and the concentration of proteases caspase-3, caspase-8, and caspase-9 were measured.

#### 3.3.7. Measurements of Cardiomyocytes’ Activity

Cardiomyocytes were inoculated into 96-well plates at 100 µL per well, and 20 µL MTT (5 mg/mL) was poured into each well. After culturing in a CO_2_ incubator at 37 °C for 4 h, 100 µL DMSO was added to each well. The 96-well plates were shaken for 15 min. The optical density (OD) of cardiomyocyte samples was measured at 570 nm [29]. Cardiomyocyte activity (%) = (OD value of test group/OD value of blank control group) × 100%.

#### 3.3.8. Measurements of ROS Activity

The cell culture medium was removed from the 96-well plates. Then, 500 µL DCFH-DA (10 µmol/mL) was added to each well. The 96-well plates were placed in an incubator at 37 °C (containing 5% CO_2_) for 20 min. After cultivation, the cardiomyocytes were washed with DMEM (excluding FBS) 3 times to fully remove the DCFH-DA that did not enter the cells. Flow cytometry was used to detect ROS in the cardiomyocytes [30].

#### 3.3.9. Measurements of Caspase-3, Caspase-8, and Caspase-9 Activity in Cardiomyocytes

The cardiomyocytes were centrifuged at 106× *g* for 10 min after trypsin digestion (0.1%). Cell lysis buffer (30 µL) was then added to each well and placed in ice water for 3 min. The mixtures were centrifuged at 6797× *g* for 10 min. The supernatant was collected and operated according to the kit’s instructions to determine the activities of caspase-3, caspase-8, and caspase-9 in cardiomyocytes [31]. Caspase activity = OD_405 nm_/OD_595 nm_.

#### 3.3.10. Measurements of Cardiomyocytes’ Apoptosis

Flow cytometry (BD FACS Aria II, Franklin, NJ, USA) was used to measure the cardiomyocyte apoptosis of mice [32]. The cardiomyocytes were washed with PBS twice and centrifuged at 238× *g* for 8 min. Cardiomyocyte precipitation was then configured with PBS to form mice cardiomyocytes suspension at a concentration of 2 × 10^5^ mL^−1^. The cardiomyocytes suspension (100 µL) was added to a Falcon test tube. Then, 500 µL of binding buffer, 5 µL of annexin V-FITC, and 5 µL of propidium iodide (PI) were added to the tube. The Falcon test tubes were mixed and stored in a dark place at 25 °C for 10 min. The cardiomyocyte apoptosis of mice was determined with 400 µL of PBS buffer in Falcon test tubes using flow cytometry.

### 3.4. Statistical Analysis

The software SPSS 20.0 (IBM, Chicago, IL, USA) was used for the ANOVA analysis of the samples in our study. Significant differences were determined by Duncan’s multiple comparison test (*p* < 0.05).

## 4. Conclusions

In this study, the ultrasonic-assisted enzymolysis extraction of FPMB was investigated along with the protective effect of FPMB on H_2_O_2_-induced injured cardiomyocytes. In the ultrasonic-assisted enzymolysis extraction of the FPMB test, the influence degree of every single factor on the extraction rate was successive: cellulase concentration > ultrasonic power > enzymolysis temperature = ultrasonic-assisted enzymolysis time. In the range of every single factor, the concentration of cellulase and ultrasonic power significantly influenced the extraction rate of FPMB. The effects of enzymolysis temperature and ultrasonic-assisted enzymolysis time on the extraction rate of FPMB were not significant.

By comparing the peak retention time of the FPMB sample with that of the mixed standard HPLC chromatograms, we found that the main flavonoids in FPMB are rutin, cynarin, and luteolin. The balance of ROS production is disrupted when cardiomyocytes are subjected to emergency oxidative damage. The proteases caspase-3, caspase-8, and caspase-9 are closely related to apoptosis and produced in large quantities. The activity of mice cardiomyocytes increased significantly after FPMB exposure, whereas the ROS content and caspase-3, caspase-8, and caspase-9 activities of mice cardiomyocytes were significantly reduced. The apoptosis rate of mice cardiomyocytes also decreased gradually; however, with the increase in FPMB dose, the apoptosis rate for cardiomyocytes was close to that for the control group. These experimental results showed that FPMB has a protective effect on the injured cardiomyocytes induced by H_2_O_2_.

## Figures and Tables

**Table 1:** Effect of cellulase mass percentage and hydrolysis temperature on flavonoids extraction rate.

Groups		Extraction Rate (%)
Cellulase mass percentage (%)	0.5	1.80 ± 0.07 ^d^
	1.0	2.63 ± 0.12 ^c^
	1.5	3.52 ± 0.15 ^b^
	2.0	5.81 ± 0.26 ^a^
	2.5	5.83 ± 0.23 ^a^
	3.0	5.92 ± 0.19 ^a^
Hydrolysis temperature (°C)	30	3.75 ± 0.11 ^c^
	35	4.82 ± 0.26 ^b^
	40	5.63 ± 0.28 ^a^
	45	5.65 ± 0.26 ^a^
	50	5.58 ± 0.19 ^a^
	55	5.49 ± 0.15 ^a^

Results are mean ± standard deviation. Different lowercase letters in the same column within the same sample express significant differences (*p* < 0.05).

**Table 2:** Effect of ultrasonic power and time on flavonoids extraction rate.

Groups		Extraction Rate (%)
Ultrasonic power (W)	200	3.69 ± 0.11 ^c^
	250	5.07 ± 0.15 ^b^
	300	6.15 ± 0.23 ^a^
	350	6.10 ± 0.18 ^a^
	400	5.91 ± 0.21 ^a^
	450	5.85 ± 0.17 ^a^
Ultrasonic time (min)	20	4.27 ± 0.16 ^c^
	30	5.12 ± 0.26 ^b^
	40	6.06 ± 0.21 ^a^
	50	6.17 ± 0.31 ^a^
	60	6.15 ± 0.16 ^a^
	70	6.23 ± 0.22 ^a^

Results are mean ± standard deviation. Different lowercase letters in the same column within the same sample express significant differences (*p* < 0.05).

**Table 3:** The results of the orthogonal test.

Number	Cellulase Mass Percentage A (%)	Hydrolysis Temperature B (°C)	Ultrasonic Power C (W)	Ultrasonic Time D (S)	Extraction Rate (%)
1	1.5	35	250	30	5.62
2	1.5	40	300	40	5.78
3	1.5	45	350	50	5.95
4	2.0	35	300	50	5.86
5	2.0	40	350	30	6.28
6	2.0	45	200	40	6.03
7	2.5	35	350	40	6.31
8	2.5	40	200	50	5.97
9	2.5	45	250	30	6.15
K_1_	17.350	17.790	17.620	18.050	
K_2_	18.170	18.030	17.790	18.120	
K_3_	18.430	18.130	18.540	17.780	
k_1_	5.783	5.930	5.873	6.017	
k_2_	6.057	6.010	5.930	6.040	
k_3_	6.143	6.043	6.180	5.927	
R	0.360	0.113	0.250	0.113	

**Table 4:** The results of the variance analysis of the orthogonal test.

Variance Source	Square Sum	Degrees of Freedom	Mean Square	F-Value	Significance
A	0.212	2	0.106	10.406	*
B	0.020	2	0.010	1.000	
C	0.160	2	0.080	7.848	*
D	0.021	2	0.011	1.056	
Error	0.020	2	0.010		

Note: F_0.01 (2,2)_ = 5.00; F_0.05 (2,2)_ = 17.00. An asterisk (*) indicates significant difference with *p* < 0.05.

**Table 5:** Effect of FPMB on the apoptosis of mice cardiomyocytes injured by H_2_O_2_.

Groups	Apoptosis Rate (%)
Control	24.20 ± 0.67 ^e^
H_2_O_2_	61.23 ± 1.81 ^a^
FPMB-L	40.80 ± 1.07 ^b^
FPMB-M	30.10 ± 0.88 ^c^
FPMB-H	26.60 ± 0.53 ^d^

Results are mean ± standard deviation. The total number of mice was 12. Different lowercase letters in the same column within the same sample express significant differences (*p* < 0.05).

## Data Availability

The data generated during the present study are available from the corresponding author on reasonable request.

## Supplementary Materials

The following are available online. Figure S1: Standard curve of rutin, Table S1: Data used to generate Table 1 and Table 2.

## Sample Availability

Samples of the compounds such as FPMB are available from the authors.
