# Supplementary material for: Ultrasonic-Assisted Enzymolysis Extraction and Protective Effect on Injured Cardiomyocytes in Mice of Flavonoids from Prunus mume Blossom
Source: Molecules. 2021 Sep 25;26(19):5818. doi: 10.3390/molecules26195818 (PMC8510299; doi:10.3390/molecules26195818)
Supplement: Supplementary file 1 [file molecules-26-05818-s001.zip › molecules-1350186-supplementary.pdf]

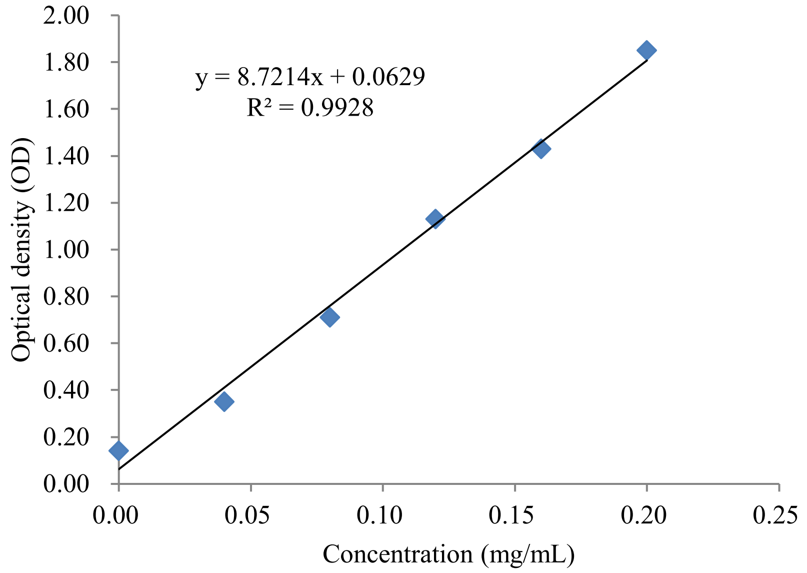

Figure S1. Standard curve of rutin.

Table S1. Data used to generate Tables 1 and 2.

Cellulase mass percentages (%)	Extraction rate 01 (%)	Extraction rate 02 (%)	Extraction rate 03 (%)	Average (%)	Standard deviation
0.5	1.74	1.87	1.78	1.80	0.07
1	2.5	2.73	2.67	2.63	0.12
1.5	3.69	3.4	3.48	3.52	0.15
2	6.09	5.57	5.78	5.81	0.26
2.5	6.08	5.62	5.78	5.83	0.23
3	6.09	5.71	5.95	5.92	0.19
Hydrolysis temperature (℃)					
30	3.74	3.86	3.65	3.75	0.11
35	5.1	4.76	4.59	4.82	0.26
40	5.93	5.39	5.56	5.63	0.28
45	5.93	5.43	5.58	5.65	0.26
50	5.79	5.51	5.44	5.58	0.19
55	5.66	5.42	5.38	5.49	0.15
Ultrasonic power (W)					
200	3.82	3.61	3.65	3.69	0.11
250	5.22	5.06	4.93	5.07	0.15
300	6.42	6.01	6.03	6.15	0.23
350	6.3	5.96	6.03	6.10	0.18
400	6.11	5.7	5.93	5.91	0.21
450	6	5.88	5.66	5.85	0.17
Ultrasonic time (min)					
20	4.45	4.16	4.19	4.27	0.16
30	5.41	4.89	5.07	5.12	0.26
40	6.27	5.85	6.05	6.06	0.21
50	6.49	6.15	5.87	6.17	0.31
60	6.33	6.12	6.01	6.15	0.16
70	6.46	6.21	6.03	6.23	0.22
